# UK Transfusion Laboratory Collaborative: Recommended minimum standards for hospital transfusion laboratories

**DOI:** 10.1111/j.1365-3148.2009.00938.x

**Published:** 2009-08

**Authors:** B Chaffe, J Jones, C Milkins, C Taylor, D Asher, H Glencross, M Murphy, H Cohen

The Serious Hazards of Transfusion (SHOT) Adverse Incident Reporting Scheme (SHOT Annual Reports, 1996–2008) has consistently reported that 30–40% of ‘wrong blood’ event errors are due to errors originating in the hospital blood transfusion laboratory with a disproportionate number occurring outside ‘core hours'. Evidence collated from two national surveys (Summary of Two National Surveys of UK Transfusion Laboratories, http://www.ibms.org/index.cfm?method=science.transfusion_science) of UK Transfusion Laboratories has formed the basis for the following recommendations aimed at reducing blood transfusion laboratory errors by 50% by 30 September 2012.

The collaborative recommendations are intended to encourage effective and appropriate use of technology and staff in hospital transfusion laboratories within the framework of current legislative requirements. These recommendations will help hospitals and trusts in achieving the minimum standards of proficiency and practice set by The Health Professions Council (HPC) (HPC Standards of Proficiency, 2004) and as required by the UK Blood Safety and Quality Regulations (as amended) (BSQR 2005).

The collaborative recognises that not all existing hospital transfusion laboratory structures may currently meet all of these recommendations. However, hospitals and trusts will be expected to work towards these recommendations if transfusion services are reconfigured, as new posts are established or new staff members are appointed.

- It is recommended that appropriate staff currently in post who do not hold one of the qualifications listed in recommendation 3.1 must demonstrate equivalence to the learning outcomes of an Institute of Biomedical Science (IBMS) Higher Specialist Diploma in Transfusion Science or an IBMS accredited MSc in Transfusion and Transplantation to meet recommendations 3.4 and 3.6 below.
- It is recommended that appropriate staff currently in post who do not hold one of the qualifications listed in recommendation 3.2 must have their competency assessed against the learning outcomes of one of these qualifications before undertaking lone-working to meet recommendations 3.4 and 3.6 below.

## Recommendations

There are three main groups of recommendations for hospital blood transfusion laboratories:

### Staffing

1.1 The collaborative recommends that the staffing levels and skill mix will be adequate to ensure the safe and effective delivery of routine and emergency services.
1.2 The collaborative recommends that these staffing levels and skill mix will be reviewed annually and agreed through the appropriate hospital or trust governance structure.
1.3 The collaborative recommends that laboratories will have protocols in place to ensure that adequate staff numbers with an appropriate skill mix are available to match the work load and case mix during all work periods.
1.4 To help facilitate compliance with the BSQR (2005), the requirements of a quality management system will be included as part of workload and service delivery when considering 1.1, 1.2 and 1.3 above.
1.5 To help facilitate compliance with the BSQR (2005), the collaborative recommends that blood transfusion laboratory lead biomedical scientists will be excluded from the following:

- The staff establishment required for core hours service provision.
- The rota for non-core hours service provision if there is any impact on core hours availability

### Technology

2.1 The collaborative recommends that all laboratories will have full walk away automation which is in use for 24 h, 7 days a week, with bidirectional interfaces to the laboratory information system. Where the workload does not warrant such technology, e.g. hospitals with a remote and rural location performing in the order of 10 group and screens per week then the collaborative expects all reasonable measures to be taken in order to mitigate laboratory errors.
2.2 The collaborative recommends that electronic issue of red cells will be introduced where a laboratory's information system supports this procedure within the British Committee for Standards in Haematology (BCSH) Guidelines on the specification and the use of information technology systems in blood transfusion practice (2007).
The collaborative recommends that where remote issue of red cells is being considered as part of service delivery, full blood tracking will be introduced alongside automated laboratory analysers and electronic issue of red cells in order to support this.

### Training and competence

3.1 The collaborative recommends that all staff at career framework level 7 (A Career Framework for Healthcare Scientists, 2005) or above who supervise and take responsibility for work at any time within a blood transfusion laboratory will have one or more of the following qualifications or equivalent experience:

- Fellowship of the IBMS (FIBMS) by examination (Special Exam, Two-part Fellowship or Higher Specialist Diploma) in Blood Transfusion or Transfusion Science;
- MSc or FIBMS in another discipline in conjunction with an IBMS Higher Specialist Diploma in Transfusion Science;
- MSc in Transfusion and Transplantation from a university accredited by the IBMS.

3.2 The collaborative recommends that all unsupervised lone-working individuals will be registered with the HPC, and all lone-working staff whether working supervised or unsupervised will have attained one or more of the following as appropriate:

- registration via the Council for Professions Supplementary to Medicine (CPSM)/IBMS logbook in haematology and hospital-based transfusion practice;
- registration via the CPSM/IBMS logbook in blood transfusion;
- IBMS Specialist Diploma in Haematology with Hospital Transfusion Practice;
- IBMS Specialist Diploma in Transfusion Science;
- BBTS Specialist Certificate in Transfusion Science Practice.

3.3 The collaborative recommends that in order to maintain competency for non-core hours working, staff who are not permanently established in blood transfusion will complete the equivalent of 10 working days per annum supervised working in a hospital blood transfusion laboratory.
3.4 The collaborative recommends that in order to maintain competency for non-core hours working, lead biomedical scientists will complete the equivalent of 10 working days per annum, autonomous, independent or lone-working in a hospital blood transfusion department and also meet recommendation 1.5 above.
3.5 The collaborative recommends that there will be a programme of on-going training and an annual competency assessment in which all individuals working at any time within the blood transfusion laboratory will actively participate.
3.6 The collaborative recommends that a senior member of staff, as defined in recommendation 3.1, will be available to provide appropriate specialist transfusion advice during non-core hours. This may require local collaboration with other hospitals and trusts.
3.7 The collaborative recommends that all individuals will be competent in local work practices prior to participation in a shift system or if lone-working in a blood transfusion laboratory during core hours.
3.8 The collaborative recommends that any temporary staff employed to work within blood transfusion laboratories will be subject to all the recommendations contained within this report.

## Evaluation

The implementation of these recommendations will be monitored as appropriate through current Medicines and Healthcare products Regulatory Agency (MHRA, http://www.mhra.gov.uk/Aboutus/Whatweregulate/Blood/index.htm; where applicable to BSQR 2005) inspections. The impact of these recommendations on transfusion laboratory errors will be monitored by SHOT reporting via the MHRA SABRE (Serious Adverse Blood Reactions and Events, http://www.mhra.gov.uk/Safetyinformation/Reportingsafetyproblems/Blood/index.htm) reporting system.
